# Dendritic Cells and Programmed Death-1 Blockade: A Joint Venture to Combat Cancer

**DOI:** 10.3389/fimmu.2018.00394

**Published:** 2018-03-01

**Authors:** Maarten Versteven, Johan M. J. Van den Bergh, Elly Marcq, Evelien L. J. Smits, Viggo F. I. Van Tendeloo, Willemijn Hobo, Eva Lion

**Affiliations:** ^1^Laboratory of Experimental Hematology, Faculty of Medicine and Health Sciences, Vaccine and Infectious Disease Institute (VAXINFECTIO), University of Antwerp, Antwerp, Belgium; ^2^Center for Oncological Research Antwerp, Faculty of Medicine and Health Sciences, University of Antwerp, Antwerp, Belgium; ^3^Center for Cell Therapy and Regenerative Medicine, Antwerp University Hospital, Antwerp, Belgium; ^4^Laboratory of Hematology, Department of Laboratory Medicine, Radboud Institute for Molecular Life Sciences, Radboud University Medical Center, Nijmegen, Netherlands

**Keywords:** dendritic cell, programmed death-1, cancer immunotherapy, combination therapy, programmed death ligand 1/2

## Abstract

Two decades of clinical cancer research with dendritic cell (DC)-based vaccination have proved that this type of personalized medicine is safe and has the capacity to improve survival, but monotherapy is unlikely to cure the cancer. Designed to empower the patient’s antitumor immunity, huge research efforts are set to improve the efficacy of next-generation DC vaccines and to find synergistic combinations with existing cancer therapies. Immune checkpoint approaches, aiming to breach immune suppression and evasion to reinforce antitumor immunity, have been a revelation in the immunotherapy field. Early success of therapeutic antibodies blocking the programmed death-1 (PD-1) pathway has sparked the development of novel inhibitors and combination therapies. Hence, merging immunoregulatory tumor-specific DC strategies with PD-1-targeted approaches is a promising path to explore. In this review, we focus on the role of PD-1-signaling in DC-mediated antitumor immunity. In the quest of exploiting the full potential of DC therapy, different strategies to leverage DC immunopotency by impeding PD-1-mediated immune regulation are discussed, including the most advanced research on targeted therapeutic antibodies, lessons learned from chemotherapy-induced immune activation, and more recent developments with soluble molecules and gene-silencing techniques. An overview of DC/PD-1 immunotherapy combinations that are currently under preclinical and clinical investigation substantiates the clinical potential of such combination strategies.

## Introduction

Dendritic cells (DCs) are key antigen-presenting cells capable of presenting tumor antigens to T lymphocytes (1) and promoting innate immunity *via*, e.g., natural killer (NK) cells (2) and γδ T cells (3). To obtain and engineer DCs for therapeutic approaches, they can be generated *ex vivo* from multiple sources such as monocytes [monocyte-derived DCs (moDCs)] and CD34^+^ hematopoietic progenitor cells, or they can be enriched from peripheral and cord blood (4–7). Exploiting their antigen-specific and immunoregulatory qualities, DCs can be furnished with tumor antigens and other targeted molecules *via* different techniques (7–9). More than two decades after the first implementation of DCs as an immunotherapy to treat cancer (10), it can be ascertained that DC-based vaccination is safe, well tolerated, and capable of inducing antitumoral immune responses. Objective clinical responses, however, are amenable to substantial improvement (11). To date, scientists believe that the full potential of DC-based immunotherapy has not yet been reached (11–13). This is evidenced by the profound and multidimensional exploration of ways to invigorate the immunotherapeutic potential of DCs, both at the level of DC vaccine engineering and combining DC therapy with other synergistic antitumor (immuno)therapies (14–20). Core objectives of this common quest are to improve DC immunopotency to promote cytotoxic and long-lasting antitumor immunity and to overcome the tumor-mediated immunosuppressive environment (9, 20). In relation to this, interfering with immune checkpoint inhibitory pathways has been on the rise. Since its second-place ranking as a potential target for immunotherapy at the Immunotherapy Agent Workshop of the National Cancer Institute in 2007 research on the inhibitory checkpoint programmed death-1 (PD-1)/programmed death ligand (PD-L) pathway has boosted massively. Due to superior antitumor effects of anti-PD-1- and anti-PD-L1-blocking antibodies, these molecules even climbed to the first position as potential targets for immunotherapy at the 29th Annual meeting of the Society for Immunotherapy of Cancer in 2015 (21). Next to exploiting the systemic monoclonal antibody (mAB) strategy, other promising PD-1-/PD-L-targeted approaches are under development. As acknowledged for DC-based vaccination, combination strategies of PD-1-targeted inhibitors with other immune (checkpoint) modulators, cell vaccines, or standard-of-care therapies will likely hold the future (22). In this review, we discuss the role of the PD-1/PD-L pathway in DC-mediated antitumor immunity and the progress of emerging strategies combining DC-based therapy with PD-1/PD-L pathway interference.

## PD-1/PD-L in Health and Disease

The PD-1/PD-L axis is one of the most studied pathways to gain understanding of immunoregulatory signals delivered by immune checkpoint receptor/ligand interaction the past few years (23, 24). Originally discovered as a mechanism of the organism to protect itself against T cell reactions toward self-antigens, interaction of PD-1 with one of its ligands (PD-L1 or PD-L2) can induce peripheral tolerance by limiting T cell activity, contributing to protection against tissue damage in case of an inflammatory response (25), prevention of autoimmune diabetes (26), or promotion of the fetal–maternal tolerance (27). Infected and malignant cells that evade immune surveillance have been ascribed to employ the inhibitory PD-1/PD-L pathway (24). Indispensable in healthy immune responses (28, 29), overexpression or induction of PD-1 and its ligands PD-L1 and PD-L2 on both immune and target cells, has been associated with immune deficiency, such as exhausted T cells, dysfunctional NK cells, expanded functional regulatory T (Treg) cells, and immune evasion and suppression (30, 31). PD-L expression can also be indispensable for the establishment of T cell immunity in other settings (28, 29). This ambiguity could be explained by findings that PD-L2 also possesses a costimulatory role (32, 33), possibly through interaction with repulsive guidance molecule b (34). Arising from either intrinsic or adaptive immune resistance (35), PD-1 and PD-L1 surface expression or secretion in different malignancies has been mostly related to poor prognosis (36–42), although discordant data have been reported (43, 44), reflecting the need to improve our understanding of the host immune system and disease-specific microenvironment.

Inhibitory PD-1/PD-L signaling not only occurs between immune cells interacting with malignant cells, but is also effective between different immune cell types shaping the tumor immune environment. This provides a strong impetus to target this inhibitory axis to breach immune inhibition and promote durable immunity. In various solid and hematological tumors, blockade of the PD-1/PD-L1 pathway has proven to reverse this immune inhibition by restoring both antitumor function and number of tumor-infiltrating CD8^+^ effector T cells, resulting in reduced tumor size and increased overall survival (45–49). While PD-1-/PD-L-targeted research predominantly focuses on effector T cells, interest in other cell types is growing. A study in metastatic melanoma patients showed that, in addition to CD8^+^ T cells, tumor-infiltrating B cells and myeloid-derived suppressor cells (MDSCs) were increased by PD-1 therapy (50). With regard to innate immunity, it has been evidenced that also NK cells are negatively regulated by PD-1 signaling during chronic infections (*Mycobacterium tuberculosis* and HIV-1) (51, 52) and in cancer (multiple myeloma, glioblastoma multiforme, ovarian carcinoma, digestive cancers) (53–59), directly relating to NK cell cytotoxic and regulatory dysfunction, immune suppression, and poor prognosis. As for T cells, blockade of this inhibitory pathway by means of mABs could restore dampened NK cell functions, at the level of both interferon (IFN)-γ response (52) and cytotoxic capacity (57). In addition, antitumor immunity mediated by invariant NK T (iNKT) cells was also shown to be improved by blockade of the PD-1/PD-L pathway (60, 61). Expression of PD-1 is also demonstrated on γδ T cells (62) and resulted in γδ T cell exhaustion that could be overcome by administration of a blocking anti-PD-L1 antibody (63, 64). A subset of γδ T cells also expresses PD-L1 conferring them with tumor-promoting characteristics by inhibiting αβ T cells (65). Therefore, PD-L1-blocking antibodies could also restore antitumor immunity by inhibiting PD-1/PD-L1 interactions between γδ and αβ T cells. With regard to immunoregulatory cells, PD-1 is also highly expressed on Treg cells (66). As shown by Sauer et al. (67) and Francisco et al. (68), interaction between PD-1 and its ligands blocks the Akt/mTOR pathway leading to an increased FoxP3 expression, resulting in Treg cell differentiation and maintenance. Furthermore, blocking the PD-1 pathway combined with antitumor vaccination showed a significant decrease in the number of intratumoral Treg cells and reduced tumor growth (69). In addition to Treg cells, a role for the PD-1/PD-L pathway has been put forward for other regulatory cells including tumor-associated macrophages (TAMs), MDSCs, and mucosal-associated invariant T (MAIT) cells (61, 70–75). While research into the effect of PD-1/PD-L blockade in these cells is limited, preclinical anti-PD-1 therapy has been shown to reduce the number of immune suppressive TAMs and MDSCs (73) and was able to increase the IFN-γ production by MAIT cells (71), indicating the valuable effect of PD-1/PD-L blockade on immune cells beyond the immune-activating CD8^+^ CTLs.

## The Role of PD-1/PD-L in DC-Mediated Antitumor Immunity

**Figure 1:**
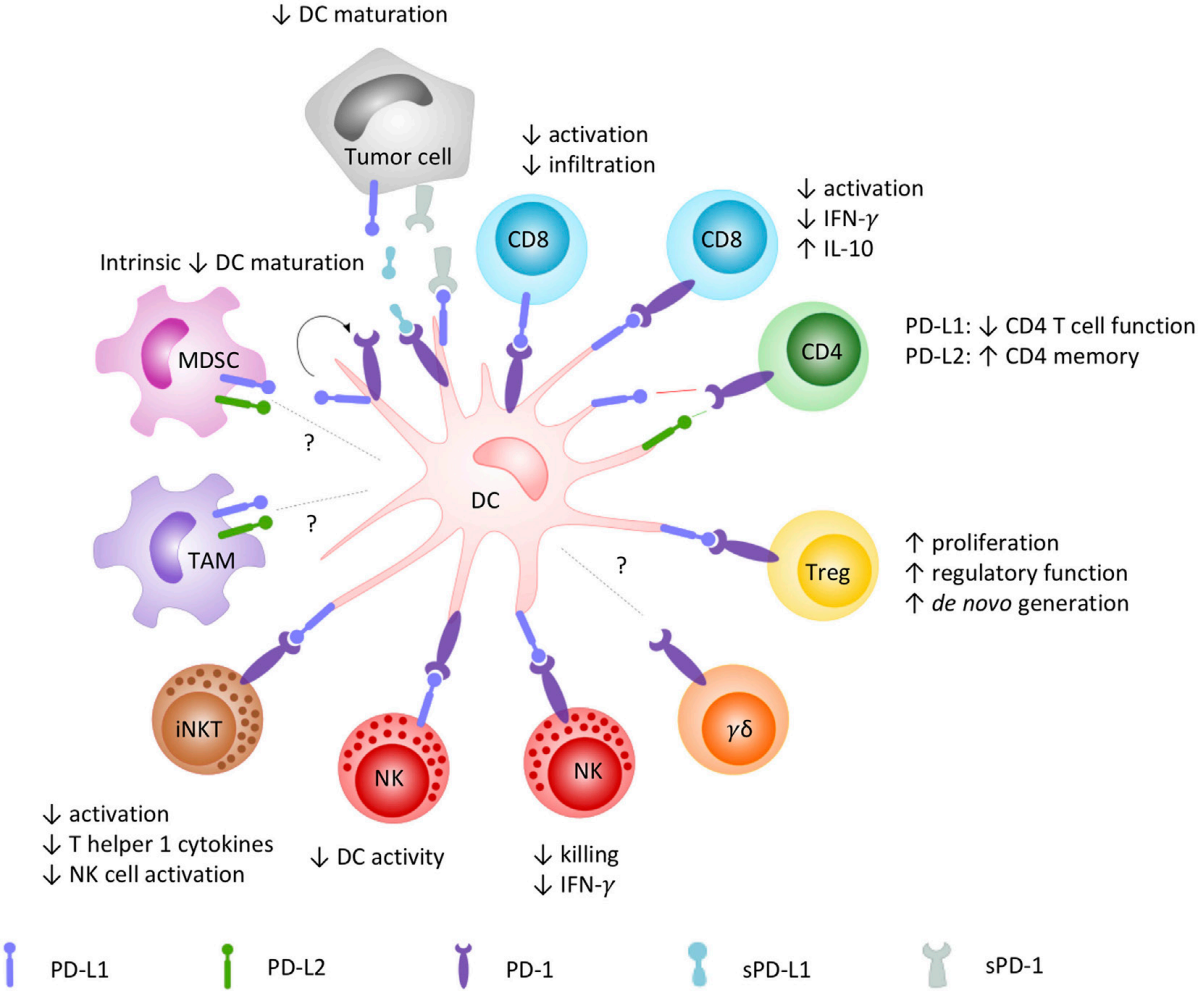
How the PD-1/PD-L signaling axis plays a role in DC-mediated orchestration of innate and adaptive immunity. DCs are renowned for their pivotal role in regulating the immune response through interaction with a variety of immune cells. DC-moderated PD-1 signaling has been demonstrated to prototypically result in an inhibitory crosstalk with effector cells, evidenced by (1) reduced infiltration and activation capacities, decreased pro-inflammatory, and increased inhibitory cytokine release by CD8^+^ and CD4^+^ T cells; (2) impaired killing, regulatory and reciprocal DC activation properties of NK cells; and (3) impaired activation, Th1-cytokine secretion, and downstream NK cell activation by iNKT cells. On the opposite, a costimulatory role for particular interactions promoting CD4^+^ T cell memory has been described as well. In crosstalk with Tregs, PD-1 engagement was shown to mediate their proliferation, regulatory function, and *de novo* generation, contributing to an immune suppressive environment. The role of PD-1-signaling in DC crosstalk with other emerging PD-1-sensitive effector (γδ T cells) and regulatory cells (MDSC, TAM) remains to be elucidated. Abbreviations: DC, dendritic cell; IFN-γ, interferon-γ; iNKT, invariant NK T cell; MDSC, myeloid-derived suppressor cell; NK, natural killer cell; PD-1, programmed death-1; PD-L1, programmed cell death ligand 1; PD-L2, programmed cell death ligand 2; sPD-1, soluble PD-1; sPD-L1, soluble PD-L1; TAM, tumor-associated macrophage; Treg, regulatory T cell.

As orchestrators of the immune system bridging innate and adaptive immunity, DCs are key players in directing antitumor immunity. Capable of expressing both the PD-1 receptor and its ligands, DCs can virtually interact with any PD-1 and PD-L-positive cell (Figure 1). In this context, the most acknowledged interaction is between DCs and T cells. PD-L surface expression on DCs [myeloid DC (mDC), plasmacytoid DC (pDC), and *in vitro* generated vaccine DC] is highest upon maturation with pro-inflammatory cytokines, Toll-like receptor (TLR) ligands, or (parts of) bacterial strains, often used to enhance the expression of costimulatory molecules on DCs (76–78). This PD-L surface expression has been demonstrated to suppress CD4^+^ and CD8^+^ T cell activity in various disease models, such as tuberculosis (79–81), HIV (82), and cancer (76, 83–88). Comparably, PD-1 expression on tumor-infiltrating mDCs has also been shown to suppress CD8^+^ T cell activity and decrease T cell infiltration in mouse models for advanced ovarian cancer (89) and hepatocellular carcinoma (90). In addition to suppression of immune activation, DC PD-L expression was also shown to be involved in the promotion of CD4^+^CD25^+^FoxP3^+^ Treg cell expansion and function (68). Tumor growth factor-beta in the tumor microenvironment promotes PD-L1 expression on DCs, further maintaining Treg cell populations (87, 91) and *de novo* generation of Treg cells (92) in favor of the immunosuppressive tumor microenvironment (84).

The role of PD-1/PD-L signaling in the crosstalk between DCs and NK cells remains largely unexplored. It has been shown that disruption of the PD-1/PD-L pathway is able to restore NK cell functions, mostly, but not exclusively in multiple myeloma (53, 55, 57, 93). Only few studies suggest a role of this pathway in DC-NK cell crosstalk and controversy remains. Ray et al. (57) demonstrated that NK cell function was abrogated by PD-L1 interactions on pDCs and PD-1 on NK cells and that NK cell functions could be restored by anti-PD-L1 treatment. On the other hand, in a preclinical mouse model, the expression of PD-L on NK cells was demonstrated to negatively regulate DC activity *via* interaction with PD-1 on DCs (94). To gain more conclusive insights in the contribution of PD-1/PD-L interactions in the crosstalk between DCs and NK cells, more research is warranted. Similar to DC-NK cell crosstalk, little is known about the role of PD-1 signaling in DC-γδ T cell crosstalk (3, 95) and how PD-1/PD-L blockade in combination with DC-based immunotherapies can further empower γδ T cells with antitumor capacities. Other innate immune cells that are able to crosstalk with DCs include iNKT cells, MAIT cells, and MDSCs (96–100). Blockade of PD-1/PD-L interactions between DCs and iNKT cells were shown to increase activation and release of T helper 1 cytokines by the latter resulting in the activation of NK cells and amplified antitumor responses (60, 101). Research on PD-1/PD-L interactions between DCs and MAIT cells or MDSCs is lacking.

Ligation of PD-1 to PD-L1/2 can also exert intrinsic effects on DCs by reverse signaling. Kuipers et al. (102) reported decreased expression of maturation markers in PD-L^+^ DCs and increased interleukin (IL)-10 production upon treatment with soluble PD-1 (sPD-1), suggesting that through reciprocal signaling a suppressive DC phenotype is attained. In another study, upregulation of PD-1 on DCs was found to be a consequence of DC maturation, especially after TLR-mediated DC activation. Blocking PD-1 during DC maturation resulted in enhanced DC survival and increased immunostimulatory properties (103). In both studies, interference with the PD-1/PD-L pathway increased the immunostimulatory properties of the DCs toward T cell activation.

The interplay of PD-1 and PD-L in DC crosstalk with (virtually all) activating and regulatory adaptive and innate immune cells impacts the productivity of antitumor immunity (Figure 1). Other than monitoring PD-L expression on tumor cells, it has been suggested that monitoring PD-L expression on infiltrating myeloid cells is more predictive for response to blockade of PD-1 signaling (104). Building on the successes of DC-based therapy (11) and PD-1-blocking strategies (105), the exploration of its combinatorial therapeutic use is rationalized to empower the clinical response rates and efficacy of these targeted approaches (7, 16).

## Strategies to Leverage DC Immunopotency by Interceding PD-1/PD-L Signaling

**Figure 2:**
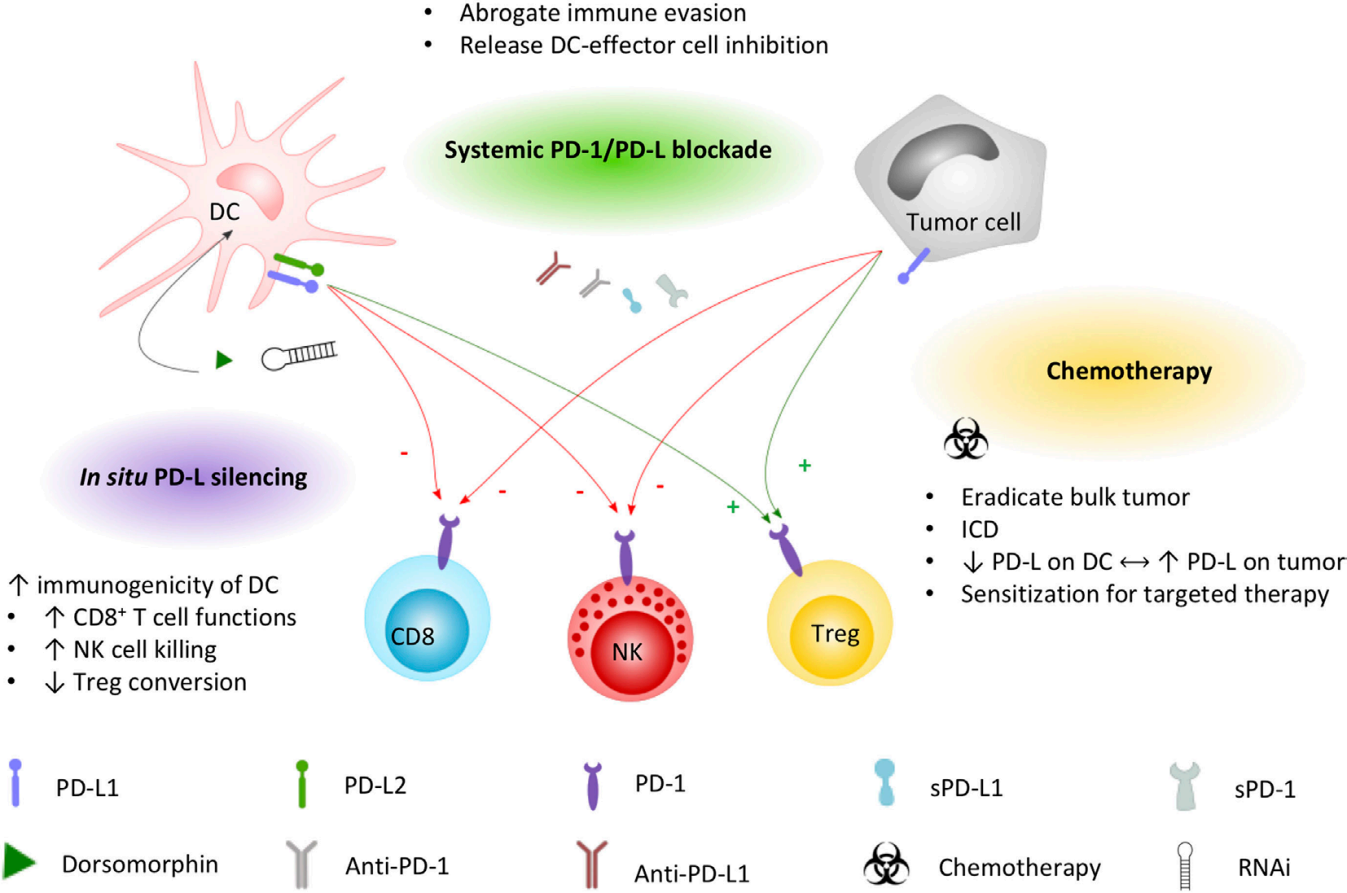
Applied strategies to leverage DC immunopotency by interfering PD-1/PD-L signaling. DC and tumor cell PD-L1 and/or PD-L2 expression exerts direct inhibitory effects (−, red arrows) on CD8^+^ T cells and NK cells, while promoting (+, green arrows) regulatory T cell functions. Current strategies to increase the immunogenicity of DC vaccines by interfering the PD-1/PD-L signaling axis include combined systemic blockade by means of PD-L1-blocking moieties. Chemotherapy triggers different mechanisms that can promote DC vaccine efficacy, including the induction of immunogenic cell death favorable for DC activation. Exploiting the PD-1 pathway, platinum-based chemotherapeutics have been demonstrated to lower PD-L expression on DCs while increasing tumor cell PD-L expression, sensitizing the tumor for systemic blockade approaches. *In situ* engineering of DC vaccines by silencing PD-L with the small molecule dorsomorphin or RNAi constructs was demonstrated to successfully improve the immunopotency of DC vaccines. Abbreviations: DC, dendritic cell; ICD, immunogenic cell death; NK, natural killer cell; PD-1, programmed death-1; PD-L1, programmed cell death ligand 1; PD-L2, programmed cell death ligand 2; RNAi, RNA interference; sPD-1, soluble PD-1; sPD-L1, soluble PD-L1; Treg, regulatory T cell.

It is generally agreed that the therapeutic potential of DC-based immunotherapy could be improved by tackling the immunosuppressive tumor microenvironment that contributes to ineffective or suboptimal responses (106, 107). Employing intrinsic and adaptive immune resistance mechanisms, PD-1 is a top-ranked checkpoint contributor to blunting immune responses. In a comprehensive review on the molecular and immunological hallmarks and prerequisites for next-generation DC vaccines, Garg et al. (20) discourses its combinatorial use with immune checkpoint inhibitors to enforce efficient antitumor activity. Based on the expression pattern of PD-1 and PD-L on immune cells and cellular contacts between DC and a myriad of immune effector and regulatory cells, blocking PD-1/PD-L interactions will likely impede tumor cell-mediated immune suppression, enhance T cell and NK cell activation and effector functions, and inhibit conversion or activation of Treg cells. However, these actions depend also on the way of implementation of PD-1/PD-L blockade with DC vaccination. Here, we elaborate on the currently applicable strategies (Figure 2) and clinical trials (Tables 1 and 2) that particularly interfere with the PD-1/PD-L pathway in the context of DC-based immunotherapies.

**Table 1:** Active clinical trials combining DC-based anticancer immunotherapy with PD-1/PD-L-targeted therapy (clinicaltrials.gov, January 14, 2018).

Intervention		Therapy schedule	Comparator(s)	Condition	Phase	*N*	Trial identifier	Status
PD-1-/PD-L-targeted therapy	Type of DC vaccine							
Anti-PD-1 Ab (nivolumab)	Autologous DC loaded with CMV pp65 mRNA	Neoadjuvant + adjuvant DC vaccination with anti-PD-1 therapy	Without neoadjuvant DC vaccination	Recurrent brain tumors	I	7	NCT02529072	Active, not recruiting

	Autologous DC loaded with NY-ESO-1 peptide	Therapy cycles of cyclophosphamide, TCR-transduced PBMC, anti-PD-1 therapy, DC vaccination, and rhIL-2	Single group	NY-ESO-1^+^ solid tumors	I	12	NCT02775292	Recruiting

	Autologous DC loaded with autologous tumor lysate	Therapy cycles of i.d. DC vaccination with anti-PD-1 therapy	DC therapy alone	Recurrent glioblastoma	II	30	NCT03014804	Not yet recruiting

Anti-PD-1 Ab (pembrolizumab)	Autologous DC loaded with peptide	Anti-PD-1 SoC post-DC therapy	Single group	Advanced melanoma	I	12	NCT03092453	Recruiting

	Autologous DC loaded with autologous tumor antigens	Therapy cycles of anti-PD-1 and cryosurgery plus i.t. DC vaccination	Single group	Non-Hodgkin lymphoma	I/II	44	NCT03035331	Recruiting

	Autologous DC	Therapy cycles of i.n. DC vaccination with anti-PD-1 therapy, radiotherapy, GM-CSF and anti-TNF-alpha therapy	Single group	Follicular lymphoma	II	20	NCT02677155	Recruiting

	DC-CIK	Therapy cycles of i.v. DC vaccination with anti-PD-1 therapy	Anti-PD-1 Ab alone	Advanced solid tumors	I/II	100	NCT03190811	Recruiting

	DC-CIK	Therapy cycles of i.v. DC vaccination with anti-PD-1 therapy	Anti-PD-1 Ab alone	NSCLC	I/II	60	NCT03360630	Recruiting

Anti-PD-1 Ab	DC-CIK	i.v. anti-PD-1 Ab-treated DC vaccination	Single group	Refractory solid tumors	I/II	50	NCT02886897	Recruiting

Anti-PD-1 Ab (CT–011)	DC/tumor cell fusion vaccine	Therapy cycles of anti-PD-1 therapy with DC vaccination post-auto-SCT	Anti-PD-1 Ab alone	Multiple myeloma	II	35	NCT01067287	Active, not recruiting

SoC CPI therapy	Autologous TLPLDC vaccine	DC vaccination (tumor lysate + yeast cell wall particles + DC) following CPI monotherapy (*comparison based on response to CPI therapy*)	CPI non-responder, progressive disease following initial response to CPI, stable disease after CPI	Metastatic melanoma	I/II	45	NCT02678741	Recruiting

Anti-PD-L1 Ab (avelumab)	Autologous DC vaccine	Therapy cycles of DC vaccination with anti-PD-L1 therapy	Single group	Metastatic colorectal cancer	I/II	33	NCT03152565	Not yet recruiting

Anti-PD-L1 Ab (durvalumab)	DC/AML fusion vaccine	*Not specified*	DC therapy alone, traditional care	Acute myeloid leukemia	II	105	NCT03059485	Recruiting

PD-L siRNA lipofection of the DC vaccine	MiHa-loaded DC	Post-allo-HSCT	Single group	Hematological malignancies	I/II	10	NCT02528682	Recruiting

*AML, acute myeloid leukemia; CPI, checkpoint inhibitor therapy; CIK, cytokine-induced killer cells; DC, dendritic cell; HSCT, hematopoietic stem cell transplantation; IL-2, interleukin 2; i.d., intradermal; i.n., intranodal; i.t., intratumoral; i.v., intravenous; MiHa, minor histocompatibility antigens; NSCLC, non-small-cell lung cancer; PBMC, peripheral blood mononuclear cells; PD-1, programmed death-1; PD-L1, programmed death ligand 1; siRNA, small interfering RNA; SoC, standard of care; TCR, T cell receptor; TLPLDC, tumor lysate particle-loaded dendritic cell*.

### Systemic Receptor-Ligand Blockade

The use of mABs that block immune checkpoints, particularly cytotoxic T lymphocyte antigen-4 (CTLA-4), PD-1, and PD-L1, has made a profound impact in the field of cancer immunotherapy (108). As of 2011, treatment of several malignancies with anti-CTLA-4- (ipilimumab), anti-PD-1- (nivolumab and pembrolizumab), and anti-PD-L1- (atezolizumab, durvalumab, and avelumab) blocking antibodies has been approved by the US FDA and EMA and a series of new inhibitors is being assessed in late stage clinical trials (105). With the experience that anti-CTLA-4 therapy comes with higher toxicity and lower response rates (16, 109, 110), the focus of research is propelling toward the PD-1/PD-L pathway as evidenced by the myriad of publications on fundamental, preclinical, and clinical PD-1/PD-L research and on its prognostic and predictive biomarker value. As an example, one of the latest developments is to extend the systemic antibody-blocking function with antibody-dependent cellular cytotoxicity (ADCC) properties. The majority of mABs bear a mutation in their Fc portion, making target cells insensitive to ADCC mediated through the FcγRIIIa on NK cells. Keeping the Fc part not mutated, avelumab resulted in ADCC-mediated clearance of PD-L1^+^ tumor cells (111).

In combination with DC vaccination, systemic blockade with anti-PD-1 mABs (112, 113) or anti-PD-L mABs (114–116) resulted in increased activation of cytotoxic CD8^+^ T cells and decreased Treg cell numbers (112) and showed better therapeutic efficacy compared with either monotherapy by preventing tumor growth and prolonging survival in tumor-bearing mice [glioblastoma (113), breast cancer (114), and melanoma (116)]. Recent studies evaluated the effect of different immune checkpoint inhibitors on human T cell responses after co-culture with allogeneic moDCs. In this setting, PD-1 and B and T lymphocyte attenuator (BTLA)-blocking antibodies could increase IFN-γ production and proliferation by T cells. Combined with anti-PD-1, other emerging immune checkpoint inhibitors such as anti-T cell immunoglobulin and mucin-domain containing-3 (TIM-3), anti-lymphocyte activating gene-3 (LAG-3), anti-CTLA-4, and anti-BTLA were able to further increase the IFN-γ-producing and proliferative capacity of T cells, while ineffective on their own (117, 118). These findings further underscore the strength of the PD-1-/PD-L-signaling axis relative to other immune checkpoint pathways.

Over the past 8 years, a select number of phase I/II clinical trials combining DC vaccination with anti-PD-1 or anti-PD-L1 antibodies in a range of malignancies have been initiated and are currently all ongoing (Table 1). With the first clinical results expected in the near future, the challenges of conceptualization of such combination therapy are already subject of discussion (20). The growing portfolio of both next-generation DC vaccines and available PD-1 and PD-L targeting mABs makes the possible treatment regimens infinite. Moreover, knowledge is growing that tumors are differentially sensitive to either DC therapy or antibody-mediated checkpoint blockade, either intrinsically or dependent on the stage of the disease. While DC-mediated therapy is consistently proven safe (7), systemic mAB therapy has to deal with several immune-related adverse effects such as skin and mucosal irritation, diarrhea, hepatotoxicity, and endocrinopathy (110, 119). Today, we are learning how to recognize and manage immune-related adverse events and toxicities and gaining knowledge on which therapeutic combinations could be applied best at what time point (120, 121). As an alternative to human(ized) mABs, different blocking moieties with advanced target specificity and affinity and reduced toxicity profiles are under investigation, including chimeric fusion proteins (AMP-224, extracellular domain of PD-L2, and an Fc portion of IgG) and nanotechnologies [nanoparticles (122) and nanobodies ((123), Theravectys, Ablynx)]. Although research in this area is limited, these alternative blockers have interesting features because of their size, stability, and pharmacodynamical properties (124), which might pave the way for implementation in combination therapy with DCs.

### Soluble PD-(L)1

Comparable to the systemic antibody approach is the use of sPD-1 receptor, which only contains the extracellular domain of the PD-1 molecule and can ligate to PD-Ls, making them inaccessible for interaction with PD-1 molecules on immune effector cells. Binding of sPD-1 to surface PD-L on DCs was demonstrated to enhance proliferation of lymphocytes *in vitro*. In addition, after administration of a vector encoding for sPD-1, tumor growth was inhibited or delayed in a murine model of hepatocarcinoma (125). Similar results were found by Song et al. (126) who additionally demonstrated increased expression of activation markers on DC in mice treated with sPD-1. Kuipers et al. (102), however, demonstrated a decrease in the expression of maturation markers on DCs treated with sPD-1. These discrepancies might be ascribed to different experimental settings such as the use of other sPD-1 encoding vectors. Applying the sPD-1 approach in human moDCs, Pen et al. (127) transfected mRNA encoding for sPD-1 or sPD-L1 in DC for transient local expression, thereby limiting possible adverse effects seen with systemic PD-1/PD-L blockade. With this approach, they demonstrated an upregulation of CD80 on sPD-1- or sPD-L1-expressing DCs and an increase in both CD4^+^ and CD8^+^ T cell effector functions without influencing the induction of Treg cells. Today, clinical trials evaluating this approach have not been registered.

### Chemo-Immunotherapy

Anticancer chemotherapeutics remain an important systemic treatment modality to arrest or eliminate rapidly growing cancer cells. Besides lowering the tumor burden, evidence is growing that these cytotoxic drugs also rely on several off-target immunological effects, including enhancement of the immunogenicity of malignant cells and, at least for some chemotherapeutics, suppression of inhibitory mechanisms (128, 129). Complementing conventional chemotherapy regimens with DC-targeted immunotherapy is therefore a promising strategy, actively investigated in clinical trials for a range of malignancies (>140 registered trials at Clinicaltrials.gov based on “DC and chemo” search). DC vaccine efficacy can avail from chemotherapy-induced immunogenic tumor cell death that facilitates an adaptive immune response specific for dead cell-derived antigens (130). In the context of immune checkpoint inhibition, the clinically established class of platinum-based chemotherapeutics has been designated to act *via* the PD-1 signaling pathway. In addition to DNA-interfering properties, oxaliplatin, cisplatin, and carboplatin were shown to inhibit the STAT6-pathway that is responsible for the upregulation of PD-1 ligands, leading to downregulation of PD-L1 and PD-L2 on both moDCs and tumor cells (131). The combination of platinum-based chemotherapeutics and DCs boosted *in vitro* T cell proliferation and enhanced T cell IFN-γ and IL-2 production (131). In other studies, however, platinum-based chemotherapeutics were reported to promote PD-L expression on blood DC subsets (132) and tumor cells (133). Enhanced PD-L expression on DCs resulted in impaired T cell activation, rationalizing that the chemotherapy effect likely depends on environmental cues, such as TLR expression on those DC subsets (132). In hepatocarcinoma cells, cisplatin promoted PD-L1 overexpression both *in vitro* and *in vivo*, suggesting a mechanism of chemotherapy resistance eventually leading to a suboptimal clinical effect of cisplatin treatment (133). The contradictory outcomes of these studies highlight the need for further research on the effect of platinum-based chemotherapeutics on the functionality of different immune cells, as well as on tumor cells of various origins. In addition, it will be interesting to extend research to the clinic to determine the optimal treatment schedule where chemotherapy and DC vaccination are combined. Such combination therapies are listed in Table 2. Although these studies are not yet completed, a pilot study on the immunogenicity of DC vaccination during adjuvant platinum-based chemotherapy in colon cancer patients demonstrated enhanced antigen-specific T cell responses after combinatorial treatment (134).

**Table 2:** Clinical trials combining DC vaccination strategies with PD-1-/PD-L1-modulating chemotherapeutics (clinicaltrials.gov, January 14, 2018).

DC-based therapy	PD-1-/PD-L-modulating chemotherapy	Indication
Autologous DC loaded with TAA-coding RNA(s)	Cisplatin	Melanoma (NCT02285413), malignant pleural mesothelioma (NCT02649829)
Autologous DC loaded with tumor lysate		Multiple myeloma (NCT00083538), ovarian cancer (NCT02432378)
Autologous DC-CIK		Esophageal cancer (NCT01691625, NCT02644863), NSCLC (NCT02651441)
DC-CTL		NSCLC (NCT02766348)

Autologous DC loaded with TAA(s)	Oxaliplatin (as part of FOLFIRINOX)	Pancreatic cancer (NCT02548169), colorectal neoplasms (NCT01413295, NCT02503150)
Autologous DC-CIK		Gastric cancer (NCT02504229, NCT02215837), colorectal cancer (NCT02202928, NCT02415699)

Autologous DC	Carboplatin	NSCLC (NCT02669719), breast cancer (NCT03387553)

*CIK, cytokine-induced killer cell; DC, dendritic cell; DC-CTL, dendritic cytotoxic lymphocyte; NSCLC, non-small-cell lung cancer; TAA, tumor-associated antigen*.

### DC-Targeted PD-L RNA Interference (RNAi) Technology

Taking into account the orchestrating role of DCs, targeted downregulation of PD-L expression on DCs is expected to potentiate DC-mediated T cell and NK cell activation and prevent Treg cell stimulation. RNAi approaches targeting immunosuppressive factors in DCs have been applied to improve immunogenic functions of next-generation DC vaccines (13). This strategy aims at enhancing DC-mediated antigen-targeted T cell responses at the level of the DC/effector cell immunological synapse, irrespective of tumor PD-L expression. Analogous to DCs expressing sPD-1 or sPD-L1 (*vide supra*), this technique offers attractive safety considerations compared to systemic antibody administration. The targeted nature of this approach shifts the *in situ* balance between immune stimulatory and inhibitory signals in the DC/effector cell immunological synapse toward immune stimulation, which has been suggested to result in reversal of the PD-1-mediated T cell exhaustion status (135).

Various preclinical studies demonstrated feasibility and effectivity of introducing small interfering RNAs or short hairpin RNAs interfering with inhibitory immune-related pathways in DCs, such as suppressor of cytokine signaling (136), indoleamine 2,3-dioxygenase (137), and PD-L1/PD-L2 (138–142). Focusing on the PD-1/PD-L pathway, silencing of PD-L1 and/or PD-L2 in DCs has been evaluated with different RNAi introduction techniques, including viral transduction and non-integrating electrotransfection, lipid nanoparticle transfection, and the cGMP-compliant transfection reagent SAINT-RED (77, 138, 141, 143, 144). Preclinical data demonstrated that PD-L-silenced DCs could (1) increase expansion, promote pro-inflammatory cytokine secretion and degranulation, and augment antitumor function of antigen-specific CD8^+^ T cells in human *in vitro* models (138, 140, 142) and (2) induce significant antitumor immunity *in vivo* in different malignant mouse models (139, 141). Alternatively, *in situ* PD-L silencing can also be achieved through the use of small molecules. Dorsomorphin, a small molecule inhibitor of the bone morphogenic protein signaling pathway, was shown to efficiently downregulate PD-L1 and PD-L2 expressions on treated DCs resulting in increased T cell proliferation and enhanced NK cell-mediated killing of target cells (145).

Today, few DC-associated RNAi approaches are currently being tested in early-phase clinical trials, including one trial evaluating PD-L1/2-silenced DC vaccines (NCT02528682). Results of this trial are awaited.

## Clinical Trials

Based on the general appreciation that DC vaccination can be improved by blockade of the PD-1/PD-L pathway, as shown by both *in vitro* experiments and *in vivo* animal models, most of these combination approaches are embedded in various clinical trials (146). With the exception of sPD-1, autologous DC vaccines are combined with (i) systemic mABs targeting PD-1 or PD-L1, (ii) platinum-based chemotherapeutics, and (iii) *in situ* PD-L RNAi to treat patients with both hematological cancers [multiple myeloma, acute myeloid leukemia (AML)] and solid tumors (renal cell carcinoma, mesothelioma, lymphoma, colon cancer, melanoma, ovarian cancer, pancreatic cancer, nasopharyngeal cancer, and glioblastoma). Clinical trials combining DC vaccination with PD-1/PD-L interference, registered by January 2018, are listed in Tables 1 and 2 and discussed in the corresponding paragraphs. The fast-growing number of clinical studies combining DC-based therapy with PD-1/PD-L blockade strategies emphasizes the potential of this combinatorial approach in the future treatment of cancer patients.

## Future Perspectives

Multimodality strategies striving to maximize the efficacy of DC-based cancer immunotherapy are emerging (16, 20, 107). Evidenced by a growing body of preclinical and clinical data, engineering next-generation DC vaccines and redirecting the tumor microenvironment are highly promising (7). The significant role of PD-1-signaling in DC-mediated antitumor immunity rationalizes its therapeutic combinatorial use in the rapidly evolving cancer immunotherapy landscape. The PD-1-/PD-L-blocking industry—and the immune checkpoint industry in general—has expanded drastically in the last years. Leading pharmaceutical companies are putting huge efforts in the development of systemic antibody therapies, with an estimated market value of $35 billion (147). The market for DC-based therapies is as big, with approximately 500 clinical trials registered evaluating DC vaccines, reflecting the immense scientific and pharmaceutical impact of such combinatorial therapy. The growing understanding of the immunological effects of some conventional chemotherapeutics, related to DC activation and PD-1 therapy sensitivity and resistance, provides rationale for the development of synergistic adjuvant combinations and carefully designed chemoimmunotherapy schedules that aim beyond the mere elimination of the suppressive tumor (20, 107). In addition to the pioneering CTLA-4 and PD-1 inhibitors, other immune checkpoints have been attributed to hamper DC-mediated immunity, including LAG-3 and TIM-3 (56, 119, 148). The LAG-3 mAB IMP321 was demonstrated to induce DC maturation (149–151) and is now further tested in clinical trials (NCT00351949, NCT00349934). TIM-3, present on, among others, DCs, was shown to induce T helper 1 cell death when interacting with its ligand galectin-9 on T cells (119, 152), whereas dual blockade of TIM-3 and PD-1 or CTLA-4 was able to suppress tumor growth with possibility of cure in a fibrosarcoma mouse model (153). Overall, targeting multiple immune checkpoints simultaneously with DC therapy is likely to result in synergistic efficacy (107).

Designed to potentiate the patient’s own immune system, unsatisfactory DC-based therapy efficacy led to an era of meticulous vaccine and protocol optimization aiming to enhance vaccine immunogenicity (7, 20). With the approval of immune checkpoint inhibitors, the significance of simultaneously targeting the inhibitory immune mechanisms was clinically established. In search of a balanced treatment, combinatorial DC and PD-1 pathway-targeted immunotherapy has some implications. The lack of specificity of systemic immune checkpoint blockade is prone to eliciting indiscriminate immune activation, resulting in significant immune-mediated adverse reactions and immune-related adverse events. In addition to the frequently observed development of therapy resistance, vigilant immunomonitoring to elucidate these mechanisms and advance early detection is warranted (105, 154, 155). Recently, resistance to anti-PD-1 therapy has been related to disturbance of antigen presentation, DC migration, and DC maturation (156), underscoring the importance of combinatorial treatment schedules. More than 20 years of clinical testing affirms that tumor-specific DC therapy is well tolerated and safe, and overstimulation, autoimmunity, or therapy resistance has been described (11, 20). By robustly breaching PD-1-related inhibitory signaling and demasking immune evasion, DC therapy could get that extra push to prevail durable antitumor immunity while compensating for the lack of specificity of immune checkpoint blockade (107).

Taken apart, it can be concluded that DC therapy and PD-1 blocking approaches will prove best in a combinatorial setting subject to the malignancy and the disease status (157). In this perspective, the search for biomarkers predicting response to DC therapy and to PD-1 pathway blockade is imperative (20, 155, 158). Although immune checkpoint inhibition can be strikingly effective in immunogenic cancers with high mutational burden like melanoma and lung cancer, tumors with a lower number of mutations and lower immunogenicity may be inherently resistant to this form of therapy (154, 155). Complementary, DC efficacy is high for at least some tumors with low mutational burden, like leukemia (159–162) and glioblastoma (20), further emphasizing the combinatorial use of DC vaccination with PD-1-targeted strategies to improve DC performance. Exemplifying a combinatorial approach with AML, DC vaccinations are typically administered as a consolidation therapy after conventional chemotherapy, to prevent relapse by eliminating residual leukemic cells and by generating durable antileukemic immunity (159, 161, 163). A role for PD-1 after conventional leukemia therapy has been demonstrated, supported by chemotherapy-induced upregulation of PD-1 on T cells and increased T cell PD-1 expression at relapse after hematopoietic stem cell transplantation (47, 164). Therapeutically, PD-1 checkpoint blockade in AML has been suggested to relieve Treg-mediated immunosuppression (47). Empowering adjuvant DC vaccination by blocking the inhibitory PD-1 axis could alleviate DC-mediated adaptive and innate antitumor immune responses, reflecting a promising combination as a follow-up therapy.

## Conclusion

In this review, we highlighted the role of the PD-1 pathway in DC-mediated antitumor immunity. Aiming to improve DC therapy efficacy, different strategies to invigorate DC immunopotency by impeding PD-1-mediated immune regulation were discussed. From the most advanced research on therapeutic blocking antibodies, lessons learned from chemotherapy-induced immune regulation, and data from more recent developments with gene-silencing techniques, it can be concluded that combinatorial DC and PD-1 pathway-targeted therapy approaches could complement or even synergize under defined circumstances. Five years after the comprehensive review on combination therapy with DC vaccines and immune checkpoint blockade by Vasaturo et al. (107), touching upon the first few preclinical studies on PD-1 combination strategies in particular, we witness that preclinical research has expanded drastically and has been translated into a number of clinical trials. We are now awaiting the first clinical results that will substantially direct future anticancer treatment approaches.

## Author Contributions

MV, JVDB, EM, and EL wrote the paper. ES, VVT, and WH critically revised the manuscript.

## Conflict of Interest Statement

The authors declare that the research was conducted in the absence of any commercial or financial relationships that could be construed as a potential conflict of interest.
